# Confidence intervals for the between-study variance in random-effects meta-analysis using generalised heterogeneity statistics: should we use unequal tails?

**DOI:** 10.1186/s12874-016-0219-y

**Published:** 2016-09-07

**Authors:** Dan Jackson, Jack Bowden

**Affiliations:** MRC Biostatistics Unit, Cambridge, UK

**Keywords:** Confidence interval width, Quadratic forms, Statistical conventions

## Abstract

**Background:**

Confidence intervals for the between study variance are useful in random-effects meta-analyses because they quantify the uncertainty in the corresponding point estimates. Methods for calculating these confidence intervals have been developed that are based on inverting hypothesis tests using generalised heterogeneity statistics. Whilst, under the random effects model, these new methods furnish confidence intervals with the correct coverage, the resulting intervals are usually very wide, making them uninformative.

**Methods:**

We discuss a simple strategy for obtaining 95 % confidence intervals for the between-study variance with a markedly reduced width, whilst retaining the nominal coverage probability. Specifically, we consider the possibility of using methods based on generalised heterogeneity statistics with unequal tail probabilities, where the tail probability used to compute the upper bound is greater than 2.5 %. This idea is assessed using four real examples and a variety of simulation studies. Supporting analytical results are also obtained.

**Results:**

Our results provide evidence that using unequal tail probabilities can result in shorter 95 % confidence intervals for the between-study variance. We also show some further results for a real example that illustrates how shorter confidence intervals for the between-study variance can be useful when performing sensitivity analyses for the average effect, which is usually the parameter of primary interest.

**Conclusions:**

We conclude that using unequal tail probabilities when computing 95 % confidence intervals for the between-study variance, when using methods based on generalised heterogeneity statistics, can result in shorter confidence intervals. We suggest that those who find the case for using unequal tail probabilities convincing should use the ‘1–4 % split’, where greater tail probability is allocated to the upper confidence bound. The ‘width-optimal’ interval that we present deserves further investigation.

**Electronic supplementary material:**

The online version of this article (doi:10.1186/s12874-016-0219-y) contains supplementary material, which is available to authorized users.

## Background

The random-effects model [1–3] is routinely used in meta-analysis. This model involves two parameters: the average effect, *μ*, and the between-study variance, *τ*^2^. Although *μ* is of primary interest, *τ*^2^ is also important because it describes the extent to which the true effects differ. For example, a small *τ*^2^ reassures us that the studies’ true effects are similar so that *μ* adequately describes the true effect in all studies. A large *τ*^2^ however means that there are important differences between the true effects, which should ideally be explained using techniques such as subgroup analyses or meta-regression [4].

A wide variety of estimates of *τ*^2^ are available. Here we focus on a class of methods for calculating confidence intervals that we refer to as using forms of “generalised heterogeneity statistics”. This includes confidence intervals that correspond to some very popular point estimators [1, 5]. Our main reason for investigating the use of these particular methods is because, under the assumptions of the random-effects model, they are exact. This means that we can explore the use of confidence intervals with unequal tail probabilities whilst retaining the nominal coverage probability; if we instead explored the use of alternative, and approximate, methods then we would have the added complication that using unequal tail probabilities would also have implications for the actual coverage probability. Hence for other methods we would have to further explore if shorter confidence intervals were due to lower coverage probabilities rather than using better statistical methods. It is for this same reason that we do not investigate the recently proposed approximate method that uses generalised heterogeneity statistics [6]. The use of unequal tail probabilities was described in some of the previous accounts of the methods that we use [7, 8] and in other accounts it is obvious that they could have been used. Hence the use of unequal tails when calculating confidence intervals using the methods we use here is not methodologically novel, but to our knowledge this paper is the first to investigate this particular issue in detail.

Point estimates of *τ*^2^ are routinely provided by meta-analysis computer software and can be used when making approximate inferences about the average effect [1, 9]. Methods for further calculating confidence intervals for *τ*^2^ have now also become available [7, 8, 10, 11]. Unfortunately, the confidence intervals for *τ*^2^ obtained from such methods are usually very wide. This is, in part, due to the fact that there is little information about *τ*^2^ in typical meta-analyses containing a handful of studies. Despite this fundamental limitation, it is natural to consider strategies for obtaining narrower confidence intervals. In this paper we discuss a simple strategy that enables one to apportion unequal amounts of the allowed type I error rate between the tails of the confidence interval. In the context of Bayesian analyses, presenting highest posterior density regions is a way to try to obtain shorter credible intervals than those that use equal probability tails of the posterior density, although this will not be successful in every case. Figure 2 below suggests that the use of unequal tails to provide shorter confidence intervals is conceptually similar to the use of highest posterior density regions, because we use quantiles where the *Q* profile statistic is greater than the conventional 2.5 % and 97.5 % quantiles. However, since the proposed methods are not likelihood based, it is not straightforward to directly compare our methods to Bayesian approaches.

For the most part, we will focus on the Q profile method [10, 11] for calculating confidence intervals, because this is the most established method that is based on generalised heterogeneity statistics. However we will also explore the use of an alternative approach [7, 8]. Our main focus will be to assess whether the possibility of using unequal tail probabilities when computing 95 % confidence intervals of this type results in shorter intervals; if this is the case then we regard the analysis as being more informative. Although the accruement of shorter confidence intervals for *τ*^2^ is a desirable goal in its own right, we will also show how this can be useful when performing sensitivity analyses for the average effect, which is the parameter of primary interest. We will see below that substantial gains can be made by ‘spending’ the majority of the tail probability when computing the upper bounds of confidence intervals for *τ*^2^.

The length and coverage of confidence intervals is only one of many criteria for evaluating them and there is a large literature that relates to this issue. The interested reader is referred to section 9.3 of Casella and Berger [12] for an accessible introduction. In addition to discussing the length (or the size in more than one dimension), the expected length and the coverage probability of confidence intervals, Casella and Berger describe a variety of other ideas. These ideas include notions such as the ‘uniformly most accurate’ confidence interval, ‘unbiased’ confidence intervals and ‘loss function optimality’. Casella and Berger provide a variety of references and exercises, so that the committed reader may explore these issues further. They also discuss Bayesian optimality of credible intervals; another way to obtain shorter confidence intervals for *τ*^2^ is to use informative priors for this parameter [13, 14] but we will focus on classical methods.

## Methods

### The random-effects model

The random-effects model for the study effect estimates *y*_1_,…,*y*_*k*_ in a meta-analysis is usually written as

1 $$ y_{i}|\mu_{i} \sim N(\mu_{i},{\sigma^{2}_{i}}), \quad \mu_{i} \sim N(\mu,\tau^{2}).   $$

where *y*_*i*_ is the estimated effect from the *i*th study. The model contains (*k*+1) variance components: ${\sigma ^{2}_{i}}$ represents the within study variance for study *i*’s estimate (assumed fixed and known in analysis but estimated in practice) and *τ*^2^ represents the variance of the true study effects *μ*_*i*_ that are assumed to be normally distributed around an average effect *μ*. In the special case where *τ*^2^ is zero, (1) is equivalent to the standard fixed-effect (or common-effect) model, where *μ*_*i*_=*μ* for all *i*. In this case all studies are assumed to provide an estimate of the same underlying quantity.

### Making approximate inferences about the average effect

Here our focus is on methods for calculating confidence intervals for *τ*^2^ but we also briefly describe the usual mode of making approximate inference in a meta-analysis for the average effect *μ*. We return to this issue below, where we explain how our methods are useful when performing sensitivity analyses for *μ*; usually the average effect is the parameter of primary interest.

Let *w*_*i*_ = $1/{\sigma ^{2}_{i}}$ be the within-study precision of the *i*th estimated effect, *y*_*i*_. The fixed-effect estimate of *μ* and its variance are given by

2 $$ \hat{\mu} = \frac{\sum^{k}_{i=1} w_{i}y_{i}}{\sum^{k}_{i=1} w_{i}}, \quad \text{Var}(\hat{\mu}) = \frac{1}{\sum^{k}_{i=1} w_{i}}   $$

which immediately gives rise to confidence intervals and hypothesis test results. However (2) assumes that *τ*^2^=0, which is a strong assumption that is relaxed by the random-effects model. In the random-effects model we replace *w*_*i*_ with $w^{*}_{i}$ where $ w^{*}_{i} = 1/({\sigma ^{2}_{i}} + \hat {\tau }^{2}) $ in (2), where $\hat {\tau }^{2}$ is a point estimate. This method for making inferences using the random effects model is only approximate however because the uncertainty in the estimate of *τ*^2^ is not taken into account in this analysis. However in meta-analyses with many studies this approximate method is sufficiently accurate in application and is widely used with moderate or even small numbers of studies. The Hartung and Knapp modification [15, 16], which shares much in common with methods already used in particle physics [17], has been proposed to provide more accurate inference for the average effect. However this method has also recently been critiqued by Wiksten et al. [18] on the grounds that it is not always conservative compared to a fixed-effect analysis.

### Generalised heterogeneity statistics

Various forms of heterogeneity statistics have been proposed in order to provide point estimates of, and subsequently confidence intervals for, *τ*^2^. We will refer to statistics of this type as generalised heterogeneity statistics, which we will define as a statistic of the form

3 $$  Q = \sum\limits^{k}_{i=1} w_{i}({\sigma_{i}^{2}}, \tau^{2}) (y_{i} - \hat{\mu})^{2}  $$

where the weights $w_{i}({\sigma _{i}^{2}}, \tau ^{2})$ are functions of the within and the between-study variances and

4 $$ \hat{\mu} = \frac{{\sum\nolimits}^{k}_{i=1} w_{i}({\sigma_{i}^{2}}, \tau^{2}) y_{i}} {{\sum\nolimits}^{k}_{i=1} w_{i}({\sigma_{i}^{2}}, \tau^{2})}   $$

so that $\hat {\mu }$ is the weighted mean of the *y*_*i*_. The choice of the functional form of $w_{i}({\sigma _{i}^{2}}, \tau ^{2})$ determines the type of generalised heterogeneity statistic. We allow the function $w_{i}({\sigma _{i}^{2}}, \tau ^{2})$ to take any form but functions that are positive and non-increasing in both ${\sigma _{i}^{2}}$ and *τ*^2^ are most appropriate, because then the weights allocated to studies are positive non-increasing functions in the total variance $({\sigma _{i}^{2}}+ \tau ^{2})$. To date, three forms of $w_{i}({\sigma _{i}^{2}}, \tau ^{2})$ have been proposed for use in generalised heterogeneity statistics.

#### The conventional heterogeneity statistic

Cochran [19] suggested using $ w_{i}({\sigma _{i}^{2}}, \tau ^{2}) =1/{\sigma _{i}^{2}}$, where ${\sigma _{i}^{2}}$ is the *estimated* within-study variance, so that the conventional weights in a fixed-effect analysis as in (2) are used when computing *Q*. DerSimonian and Laird [1] provide the expectation of this statistic and suggested matching this expectation to the observed *Q* in order to obtain a moments based estimator of *τ*^2^. Hoaglin [20] clarifies that Cochran used the estimated within-study variances when calculating his statistic; since here we take the within-study variances as fixed and known in analysis, using $ w_{i}({\sigma _{i}^{2}}, \tau ^{2}) =1/{\sigma _{i}^{2}}$ to indicate Cochran’s heterogeneity statistic suppresses the distinction between the estimated and true within-study variances. This means describing the conventional heterogeneity statistic as Cochran’s heterogeneity statistic is not completely historically accurate. However we continue to associate Cochran with this particular heterogeneity statistic, so that his valuable contribution to meta-analysis may continue to be recognised.

#### DerSimonian and Kacker’s generalised heterogeneity statistics

DerSimonian and Kacker [21] suggested using $w_{i}({\sigma _{i}^{2}}, \tau ^{2}) = a_{i} $, where *a*_*i*_ is any fixed positive constant. Since the within-study variances are treated as fixed and known, *a*_*i*_ may be any positive function of ${\sigma _{i}^{2}}$. This includes the reciprocal function so that DerSimonian and Kacker’s suggestion includes the previous heterogeneity statistic as a special case. If all *a*_*i*_ are identical then *Q* becomes an unweighted sum of squares. Hence DerSimonian and Kacker’s generalised statistic includes the possibility of using equal weights, an idea that was also suggested by DerSimonian and Laird [1].

#### The *Q* profile heterogeneity statistic

Using $w_{i}({\sigma _{i}^{2}}, \tau ^{2}) = 1/({\sigma _{i}^{2}}+ \tau ^{2})$ provides a pivot for *τ*^2^ that can also be be used for estimation. This is a markedly different choice of weights to the previous two suggestions because the weights are now a function of the unknown parameter *τ*^2^. Hence the *Q* profile heterogeneity statistic is a function of *τ*^2^ and we emphasise this by writing this *Q* statistic as *Q*(*τ*^2^). This choice of $w_{i}({\sigma _{i}^{2}}, \tau ^{2})$ is very convenient because $Q(\tau ^{2}) \sim \chi ^{2}_{k-1}$ for all *τ*^2^. Hence solving *Q*(*τ*^2^)=*k*−1 for *τ*^2^ provides an estimate of $\hat {\tau }^{2}$ that is generally credited to Paule and Mandel [5]. *Q*(*τ*^2^) is a decreasing function in *τ*^2^ [10] so that this estimate is unique. If *Q*(0)<*k*−1, so that there is no nonnegative *τ*^2^ that satisfies *Q*(*τ*^2^)=*k*−1, then $\hat {\tau }^{2}$ is taken to be zero. It has recently been shown that the Paule-Mandel and the Empirical Bayes [22, 23] estimators are equivalent in the more general context of random effects models for meta-regression [24]. Bowden et al. [25] also noted the equivalence of the Paule-Mandel estimator and the Empirical Bayes approach of Carter and Rolph [26].

### Confidence intervals for the between-study variance

Many methods have been proposed to derive confidence intervals for *τ*^2^ but most rely on asymptotic arguments [11]. For this reason they generally fail to achieve nominal coverage, and this poor performance is exhibited in confidence intervals [25]. Several authors have proposed exact (under the random-effects model) methods for calculating confidence intervals for *τ*^2^ using various forms of generalised heterogeneity statistics. All these methods have been shown to result in confidence *intervals* rather than more general confidence sets that need not be an interval [27].

#### The *Q* profile method

Perhaps the best known method of this type is the *Q* profile method [10, 11]. As noted above, the choice of weights $w_{i}({\sigma _{i}^{2}}, \tau ^{2}) = 1/({\sigma _{i}^{2}}+ \tau ^{2})$ results in $Q(\tau ^{2}) \sim \chi ^{2}_{k-1}$. Hence *Q*(*τ*^2^) is a pivot in *τ*^2^ with a very well known distribution. Since *Q*(*τ*^2^) is decreasing in *τ*^2^ [10], we can use critical values from the $\chi ^{2}_{k-1}$ distribution, $a = \chi ^{2}_{\alpha _{2}, k-1}$ and $b = \chi ^{2}_{1-\alpha _{1}, k-1}$, where $\chi ^{2}_{\alpha, v}$ is the *α* quantile of the $\chi ^{2}_{k-1}$ distribution, to define a (1−*α*)×100 % confidence interval for *τ*^2^ where *α*_1_+*α*_2_=*α*. The values of *τ*^2^ that lie in the confidence interval satisfy

5 $$ P(a < Q(\tau^{2}) < b) = 1-\alpha   $$

If no *τ*^2^ satisfies (5), because $Q(0)<\chi ^{2}_{\alpha _{2}, k-1}$, then we can either provide a null set [11] or provide the interval [0,0]={0} [8, 10]. A Newton-Raphson method for implementing the *Q* profile method is available [27]. Throughout we use *α*_1_ and *α*_2_ to denote the tail probabilities used in the lower and upper bounds of the confidence interval for *τ*^2^, respectively.

#### Jackson’s method

Biggerstaff and Jackson [7] showed how the conventional heterogeneity statistic can be used to obtain exact (under the random-effects model) confidence intervals and Jackson [8] extended this method to use the more general heterogeneity statistics proposed by DerSimonian and Kacker [21]. Jackson [8] showed that DerSimonian and Kacker’s generalised heterogeneity statistics are distributed as a linear combinations of ${\chi ^{2}_{1}}$ random variables, where the coefficients depend on *τ*^2^, and where the cumulative distribution function of these *Q* statistics is continuous and decreasing in *τ*^2^. This means that 100(1−*α*)% confidence intervals can be obtained as the values of *τ*^2^ that provide

6 $$  P(Q\ge q) \ge \alpha_{1}  $$

and

7 $$  P(Q \le q) \ge \alpha_{2}  $$

where *q* in (6) and (7) is the observed value of a DerSimonian and Kacker generalised heterogeneity statistic. If no *τ*^2^ satisfies (7), because *P*(*Q*≤*q*)<*α*_2_ for *τ*^2^=0, then we can provide either a null confidence set or the interval [0,0] as in the *Q* profile method. Jackson [8] suggested using the weights $w_{i}({\sigma _{i}^{2}}, \tau ^{2}) =1/\sigma _{i} $ in applications where some between-study variation is anticipated but it is uncertain how much.

#### Meta-regression and other extensions

All of these methods using generalised heterogeneity statistics have been extended to the meta-regression setting [27]. To our knowledge, the functional forms of $w_{i}({\sigma _{i}^{2}}, \tau ^{2})$ are the only ones that have been considered to date. The possible use of further forms of $w_{i}({\sigma _{i}^{2}}, \tau ^{2})$ deserves further investigation.

## Results and discussion

We begin with the preliminary investigation that we performed which motivated us to carefully examine the possibility of using unequal tails when calculating confidence intervals for *τ*^2^. It has been claimed that the frequently wide confidence intervals that are obtained in practice are due to large upper bounds of confidence intervals [7, 8]. If this claim is true, then one way to obtain shorter confidence intervals, whilst retaining the coverage probability of 100(1−*α*)%, is to take *α*_2_>*α*_1_. This means that unequal probabilities are used in the two tails, where the majority of *α* is ‘spent’ in the tail of the upper bound, to reduce the upper bound and so the width of the confidence interval.

*The W-optimal interval*

In order to investigate the full potential of using *α*_2_>*α*_1_, we will focus on the *α*-split that *post hoc, minimises* the resulting Q profile confidence intervals’ width. We will return to Jackson’s method later, but we will begin with the Q profile method because this is the longer established method. For a given value of *α* (we will use the conventional *α*=0.05 throughout), we find the values $\alpha ^{*}_{1}$ and $\alpha ^{*}_{2}$, subject to the constraint that $\alpha ^{*}_{2}=\alpha -\alpha ^{*}_{1}$, such that the resulting interval from (5) with $\alpha _{1}=\alpha _{1}^{*}$ and $\alpha _{2}=\alpha _{2}^{*}$ is shorter than any other interval where *α*_1_+*α*_2_=*α*. We will refer to the interval derived in this way as ‘W-optimal’, which is an abbreviation for ‘width optimal’.

It is important to recognise that the repeated sampling properties of the methods described above assume that *α*_1_ and *α*_2_ are specified in advance. Hence the theory set out above provides no assurance that the W-optimal interval will achieve the nominal coverage probability. Indeed there is the natural suspicion that, by choosing $\alpha _{1}^{*}$ and $\alpha _{2}^{*}$ that post-hoc minimise the confidence interval width, that the W-optimal interval will possess a coverage probability that is well below the nominal. We will investigate this issue below, but for now we are content to use the mathematical definition of the W-optimal interval to explore which values of *α*_1_ and *α*_2_ would result in the shortest confidence interval had these values been specified in advance. The reader should note that, for the present, we refer to the W-optimal interval, and not the W-optimal *confidence* interval, because the repeated sampling properties of the *W*-optimal interval are not investigated until later in the paper. Furthermore, as we explain in the discussion, we suggest that further investigation is needed before we can safely recommend presenting the *W*-optimal interval as a confidence interval.

*The NSCLC4 meta-analysis*

**Fig. 1:**
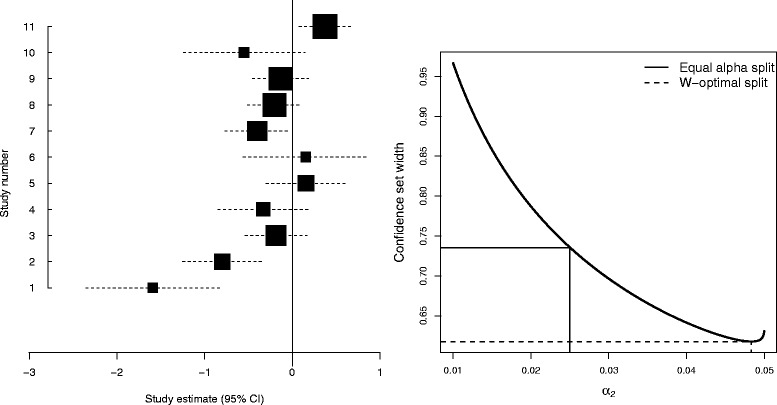
The NSCLC4 meta-analysis. *Left*: Forest plot. *Right*: Confidence interval width for *τ* ^2^ as a function of *α*_2_

Figure 1 (left) shows a forest plot of the NSCLC4 meta-analysis described in Bowden et al. [25]. The eleven RCTs making up the meta-analysis compared the effect of supportive care plus chemotherapy versus supportive care alone for patients with non-small-cell lung cancer. The results are shown on the log hazard ratio scale. Figure 1 (right) shows the 95 % confidence interval width for *τ*^2^ in the NSCLC4 meta-analysis as a function of *α*_2_. The conventional ‘equal– *α*’ approach (*α*_1_=*α*_2_=0.025) yields a confidence interval for *τ*^2^ of (0.052,0.787). The W-optimal interval for *τ*^2^ is (0.021,0.638), which is attained for $\alpha ^{*}_{1}$ = 0.2 % and $\alpha ^{*}_{2}$ = 4.8 %. In order to minimise the confidence interval’s width, we spend around 96 % of *α* on the upper confidence bound and so use *α*_2_>>*α*_1_ to obtain the shortest 95 % confidence interval.

*Further illustration of the NSCLC4 meta-analysis*

**Fig. 2:**
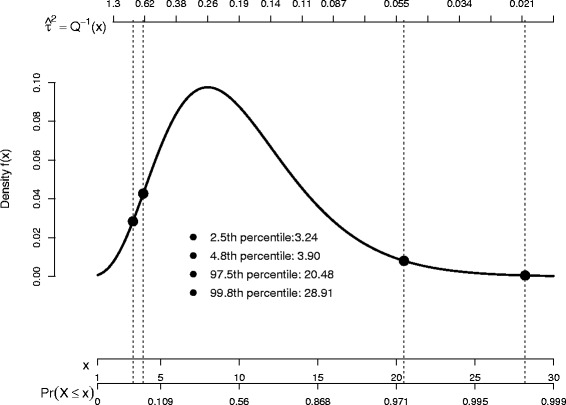
An illustration of the Q profile interval estimation for the NSCLC4 data using a confidence interval with equal tails and the ‘W-optimal’ interval

Figure 2 illustrates why *α*_2_>>*α*_1_ provides shorter confidence intervals for the NSCLC4 meta-analysis. In Fig. 2, the density *f*(*x*) of $\chi ^{2}_{k-1}$ is plotted against both the value of the random variable *x* and the corresponding value of the cumulative distribution function (shown as a proportion). Also shown on the upper horizonal axis is *Q*^−1^(*x*) for these data, where *Q*^−1^(·) is the inverse of *Q*(*τ*^2^); from (5) this function gives the confidence limits and so provides the interval estimation of *τ*^2^ for the NSCLC4 meta-analysis. The conventional 2.5 % and 97.5 % critical values, and the W-optimal critical values of $\alpha ^{*}_{2}=4.8\,$% and $1-\alpha ^{*}_{1}= 99.8\,$%, are also shown on Fig. 2 as vertical lines.

The main observation from Fig. 2 is that *Q*^−1^(*x*) is extremely non-linear in *x*. Increasing *α*_2_ from its conventional value of 2.5 %, to its optimal 4.8 %, drastically decreases the upper confidence bound shown on the upper horizonal axis, despite the fact that the *χ*^2^ percentile changes only slightly from 3.24 to 3.90. Conversely, the lower bound decreases only slightly when decreasing *α*_1_ to its optimal value, despite the fact that the *χ*^2^ quantile increases substantially from 20.48 to 28.91. Hence, as a direct consequence of the non-linear nature of *Q*^−1^(*x*), taking *α*_2_>>*α*_1_ drastically reduces the confidence interval width.

*Further trial examples*

Table 1 summarises the results obtained for the NSCLC4 meta-analysis and also shows the same results for three additional meta-analyses of cancer trials also discussed in Bowden et al. [25]. We report the *I*^2^ statistic [28] and the DerSimonian and Laird estimate of *τ*^2^ for each meta-analysis in order to quantify the heterogeneity present. The W-optimal intervals are substantially narrower in each case. Like the NSCLC4 meta-analysis, the W-optimal interval for the CERVIX1 meta-analysis involves a highly unequal ‘ *α*–split’ with *α*_2_>>*α*_1_. In the case of the NSCLC1 and CERVIX3 meta-analyses the nature of the W-optimal interval is even more extreme, where the optimal intervals are one-sided ($\alpha ^{*}_{1}=0$, $\alpha ^{*}_{2}=0.05$). The DerSimonian and Laird point estimator and the Q profile confidence interval are based on different statistical principles, so that the point estimates of *τ*^2^ in Table 1 are not guaranteed to lie within the confidence intervals. Although this only happens in rare cases, this cannot occur when the Paule-Mandel [5] point estimator is used. Hence the Paule-Mandel point estimate and the Q profile confidence interval are especially natural estimators to report in conjunction with each other.

**Table 1:** Summary of the four meta-analysis examples

Meta	k	*I* ^2^	$\hat {\tau }^{2}$	Equal- *α*	W-optimal	$\alpha ^{*}_{2}$	Width
Analysis				CI	interval		Ratio
CERVIX3	5	56 %	0.087	(0, 1.660)	(0, 1.100)	0.050	0.662
NSCLC4	11	75 %	0.132	(0.052, 0.787)	(0.021, 0.638)	0.048	0.839
NSCLC1	17	45 %	0.024	(0.000, 0.181)	(0, 0.147)	0.050	0.815
CERVIX1	18	62 %	0.112	(0.041, 0.500)	(0.017, 0.427)	0.046	0.892

*I* ^2^ is the heterogeneity statistic of Higgins and Thompson [28] and $\hat {\tau }^{2}$ is the DerSimonian and Laird estimate. In each case we show the equal tailed (*α*_1_=*α*_2_=0.025) 95 % confidence interval, the W-optimal interval, the value of $\alpha ^{*}_{2}$ that provides the W-optimal interval and the ratio of the width of the W-optimal interval and the equal tailed confidence interval. In each case we see that there is substantial reduction in the interval width by adopting *α*_2_>>*α*_1_

### Conclusions from the examples

All four examples indicate that using substantially larger *α*_2_ values can result in considerably shorter 95 % confidence intervals for *τ*^2^. In each of the four cases, the W-optimal interval results in smaller upper and lower confidence interval bounds, where the upper confidence bound is reduced very substantially but the lower bound is only reduced slightly. This suggests that we can obtain shorter 95 % confidence intervals by taking *α*_2_>>*α*_1_ in practice.

#### A final observation from the examples

One important and final observation from the examples is that, by taking *α*_2_>*α*_1_, we necessarily obtain smaller lower and upper confidence interval bounds compared to intervals using the more conventional *α*_2_=*α*_1_. This means that smaller values of *τ*^2^ are contained in *α*_2_>*α*_1_ confidence intervals, so that less heterogeneity is inferred from them.

Although we focus on the width of the confidence intervals, we feel that it is not inappropriate to also prefer the use of *α*_2_>*α*_1_ on the grounds that it reduces both confidence interval bounds. This is because the estimates of *τ*^2^ in Table 1 are very much closer to the conventional lower bounds than the upper bounds; this is quite generally the case because of the illustration provided by Fig. 2. Instead using *α*_2_>*α*_1_ reduces (but does not remove) the extreme asymmetry of confidence intervals for *τ*^2^ around the point estimate. Although approximate confidence intervals are often better calculated on the log(*τ*^2^) scale [27], which also gives rise to this type of asymmetry, they can also be computed on the *τ*^2^ scale [29] which results in symmetrical confidence intervals. Those who may find the location of point estimates within conventional confidence intervals disconcerting, and prefer presenting less asymmetric confidence intervals for *τ*^2^, are likely to also prefer to use *α*_2_>*α*_1_ on the grounds that this provides confidence intervals where the point estimates are considerably closer to the centre of the interval. We should be clear however that there is no theoretical objection to presenting point estimates that lie far away from the centre of confidence intervals, indeed point and interval estimation are two different types of statistical procedure, but we suspect that less asymmetric confidence intervals will be a desirable consequence for some applied analysts.

### Confidence intervals for the between-study standard deviation

The previous results provide some empirical evidence that notably shorter 95 % confidence intervals for *τ*^2^ can be obtained by using *α*_2_>*α*_1_. These findings also apply to confidence intervals for linear functions of *τ*^2^ but the conclusions above do not apply to non-linear functions of *τ*^2^. For example, the R *metafor* package [30] provides interval estimation for *τ*^2^, *τ*, *I*^2^ and *H*^2^. We return to the possibility of performing interval estimation for the heterogeneity statistics *I*^2^ and *H*^2^ in the discussion; whether it is appropriate or not to provide confidence intervals for these heterogeneity statistics depends on one’s willingness to accept them as functions of *τ*^2^ and so potential parameters of interest. However *τ* is clearly an interpretable parameter. In this section we examine the potential use of *α*_2_>*α*_1_ when calculating 95 % confidence intervals for *τ* but in all other sections we consider 95 % confidence intervals for *τ*^2^. Since moment-based estimates of *τ*^2^ are usually presented in application, we anticipate that most interest will lie in the width of intervals for the between-study variance. However we also examine the between-study standard deviation, in order to explore the implications of a non-linear function of *τ*^2^, because our results on the *τ*^2^ scale are not invariant to this type of transformation.

In Table 2 we show the results as in Table 1 but this time we report our findings for the *τ* scale; the W-optimal interval is defined as the shortest confidence interval as above but the W-optimal interval is now the shortest interval resulting from (5) on the *τ* scale. Table 2 shows that, for the three examples where *k*>10, the W-optimal interval on the *τ* scale is only slightly shorter than the conventional 95 % confidence interval and that the corresponding optimum $\alpha ^{*}_{2}$ is now much closer to 0.025. However for the CERVIX3 example, where *k*=5, we still have $\alpha ^{*}_{2}=0.05$ and the W-optimal interval is still substantially shorter than the conventional confidence interval. This suggests that notably shorter confidence intervals can only be obtained on the *τ* scale by taking *α*_2_>>*α*_1_ when *k* is small. Very many meta-analyses involve such small numbers of studies in practice.

**Table 2:** Summary of the four meta-analysis examples

Meta	k	*I* ^2^	Equal- *α*	W-optimal	$\alpha ^{*}_{2}$	Width
Analysis			CI (*τ*)	interval (*τ*)		Ratio
CERVIX3	5	56 %	(0, 1.287)	(0, 1.048)	0.050	0.814
NSCLC4	11	75 %	(0.227, 0.887)	(0.193, 0.824)	0.040	0.954
NSCLC1	17	45 %	(0.013, 0.426)	(0.028, 0.436)	0.021	0.986
CERVIX1	18	62 %	(0.201, 0.707)	(0.182, 0.678)	0.035	0.982

*I* ^2^ is the heterogeneity statistic of Higgins and Thompson [28]. In each case we show the equal tailed (*α*_1_=*α*_2_=0.025) 95 % confidence interval for *τ*, the W-optimal interval for *τ*, the value of $\alpha ^{*}_{2}$ that provides the W-optimal interval (also for *τ*) and the ratio of the width of the W-optimal interval and the equal tailed confidence interval. In each case we see that there is reduction in the interval width by adopting *α*_2_>>*α*_1_

### An analytical investigation

The four examples examined above suggest that shorter 95 % confidence intervals for *τ*^2^, and to a lesser extent *τ*, can be obtained by taking *α*_2_>>*α*_1_. However the results from these four examples may not generalise to other settings. Our primary proposal for investigating whether this is the case or not is the simulation study described below. However it is also possible to make analytical progress, using the artificial and special case where all studies are the same ‘size’, that is ${\sigma _{i}^{2}}=\sigma ^{2} = w^{-1}$ for all *i*. This special case has been used previously to obtain analytical results that can be used as a guide to how meta-analytic techniques perform [9, 31]. We can then define *I*^2^=*τ*^2^/(*σ*^2^+*τ*^2^) as the proportion of variation that is due to between-study variance. This means that *I*^2^ represents the true quantity that *I*^2^ statistics estimate [28] and we can interpret our findings in terms of this very popular statistic. Another advantage of exploring this simple special case is that all the methods based on generalised heterogeneity statistics reduce to the same approach in this situation, so that the conclusions from this analytical investigation apply to all the methods we discuss.

The resulting investigation is mathematically technical, and so we provide full details of this analytical work in the web Additional file 1 that accompany the paper. Briefly however, this investigation supports the conclusion that using *α*_2_>>*α*_1_ can result in markedly shorter 95 % confidence intervals for *τ*^2^ for the sample sizes usually encountered in practice (*k*≤100, say, although this is most noticeable for much smaller *k*). This analytical investigation also suggests that this is also the case for 95 % confidence intervals for *τ*, although here the gain is less substantial because the reduction in average confidence interval width is less impressive.

However the analytical investigation raises serious concerns about the widths of 95 % confidence intervals resulting from *α*_1_=0 and so *α*_2_=0.05, which in any case are at best highly undesirable because this choice necessarily results in a lower confidence interval bound of zero. Hence we are unable to rule out small values of *τ*^2^ when using such an extreme approach. This is despite the fact that *α*_1_=0 and *α*_2_=0.05 is width-optimal for two of the examples’ 95 % confidence intervals for *τ*^2^, and also for one of the examples’ 95 % confidence interval for *τ*. The analytical results shown in the Additional file 1 show that *α*_1_=0 and so *α*_2_=0.05 is width-optimal if the between-study variance is equal to zero, or if the sample size is small and the between-study heterogeneity is mild, but more generally this very extreme allocation can result in much wider 95 % confidence intervals. If *τ*^2^=0 then it is intuitively obvious that spending all the tail probability in reducing the upper bound is width-optimal, and this also appears to apply to small *τ*^2^ in small samples, where 95 % confidence intervals are necessarily wide. However *τ*^2^ is unknown and may be larger, in which case *α*_1_=0 and *α*_2_=0.05 is far from width-optimal, in addition to being unacceptable on the grounds that the lower bound is then necessarily zero.

The analytical investigation therefore supports the use of *α*_2_>>*α*_1_ as suggested by our four examples, but also strongly discourages the use of *α*_2_=0.05 despite the empirical results. Motivated by our examples and our analytical investigation we will therefore explore three possibilities in the simulation studies that follow: i) the conventional ‘equal tails approach’ *α*_1_=*α*_2_=0.025; ii) the unequal (but fixed in advance) ‘ *α*-split’ of *α*_1_=0.01 and *α*_2_=0.04; and iii) the possibility of presenting the W-optimal interval as a confidence interval. The last two possibilities are presented as possible ways to reduce the confidence interval width.

### Simulation study

As explained above, the results from our four examples and the analytical results in the Additional file 1 support the use of unequal tails when computing confidence intervals for *τ*^2^. However these results may not generalise to other settings, and there may also be adverse consequences from adopting this strategy. We will therefore perform some simulation studies to further examine the issues.

#### The optimum value of *α*_2_

**Fig. 3:**
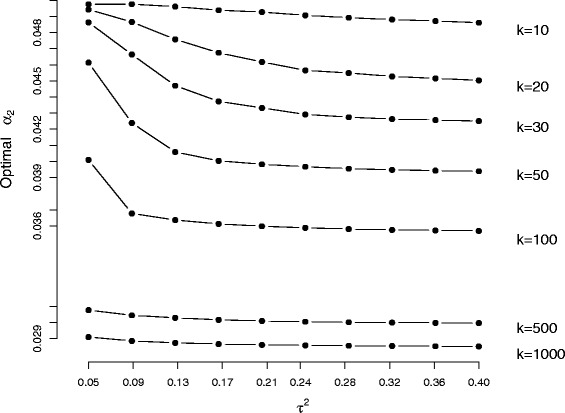
Relationship between *k*, *τ* ^2^, *I* ^2^ and the optimum $\alpha _{2}^{*}$

Here meta-analyses of *k* studies are simulated from the random-effects model (1). In each case the *σ*_*i*_ are generated from a Uniform(0.2,0.5) distribution and *μ*=0 (its value is irrelevant). The between-study variance *τ*^2^ is varied between 0.05 and 0.4, giving rise to meta-analyses with mean *I*^2^ values ranging from 30 to 75 %. These values of *τ*^2^ were chosen in order to reflect a realistic range of *τ*^2^ and *I*^2^ where the random effects model is likely to be applied in practice. Fifty thousand simulations were used in all simulation runs. Figure 3 shows how the average optimal value of *α*_2_ when calculating 95 % confidence intervals for *τ*^2^ varies as a function of study size and the amount of heterogeneity present. For realistically sized meta-analyses of less than 30 studies, the the optimum *α*_2_ lies between around 4.3 % and 5 % and is a decreasing function of *τ*^2^. Furthermore, equal– *α* splits remain sub-optimal even for fanciful meta-analyses of 1000 studies, with an average optimal *α*_2_ of around 0.03. The simulation study reassures us that the conclusions made previously generalise to other settings.

We next investigate two secondary issues: presenting the W-optimal interval as a confidence interval and investigating whether or not the same principles apply to Jackson’s [8] method. In order to keep the size of the simulation study manageable, and also investigate situations where the random-effects model is reasonably well identified but there is not an implausibly large number of studies, we restrict further investigations to *k*=15. This number of studies is half way between the two smallest sample sizes explored in Fig. 3.

#### Presenting the W-optimal interval as a confidence interval

Given the very wide confidence intervals for *τ*^2^ generally obtained in application, and the potential gain in using alternative values of *α*_2_, it is tempting to consider presenting the W-optimal interval as a confidence interval. As emphasised above, the theory described above provides no reassurance that the repeated sampling properties of the W-optimal interval make it suitable as a confidence interval. Furthermore, presenting the W-optimal interval in this way is open to criticism such as ‘cherry picking’ or ‘cheating’, because it may be seen as presenting the best results from a series of statistical analyses of the same data. However, provided it is stated in advance of looking at the data that the W-optimal interval will be presented as a confidence interval, the repeated sampling properties, and so the coverage probability, of the W-optimal interval can be assessed via simulation study. Hence we will now present the W-optimal as a confidence interval and investigate its repeated sampling properties.

**Fig. 4:**
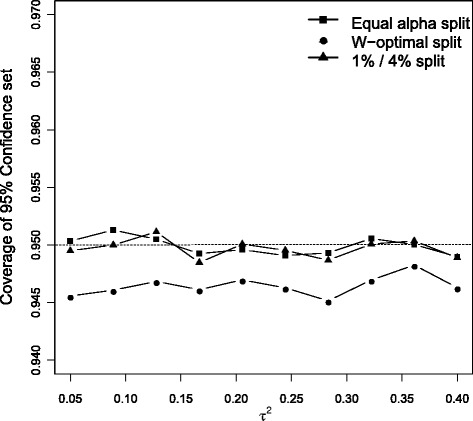
Coverage of the three confidence interval approaches

We simulate under the same data generating model for meta-analyses as in the previous section but now with *k*=15 studies. For each value of *τ*^2^ we calculate the empirical coverage of the three ways of choosing *α*_1_ and *α*_2_. The estimated coverage probabilities of these three approaches are shown in Fig. 4. As dictated by theory, the conventional Q profile method (*α*_1_=*α*_2_=0.025) maintains the nominal coverage across all simulation scenarios. Also as dictated by theory, the unequal but fixed *α*-split of *α*_1_=0.01 and *α*_2_=0.04 also has the correct coverage. The *W*-optimal approach, as might be expected, has a coverage below the nominal level. However, its sub-optimality is very small (of the order of 0.5 %).

**Fig. 5:**
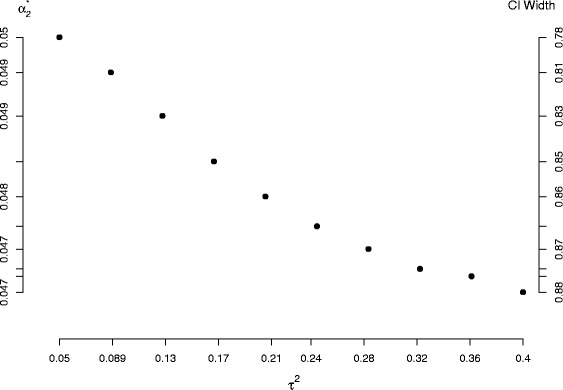
Pattern of variation in the mean values of other pertinent simulation outputs

The simulation study suggests that the coverage probability of the W-optimal interval may be sufficiently good to present this interval as a confidence interval. However there are two important caveats. Firstly, further investigation is needed into its use before it can be safely recommended. Secondly, if the W-optimal interval were to be presented as a confidence interval, it would be important to present it as such rather than leave it unclear whether or not the *α* split was specified in advance. Figure 5 highlights how the average confidence interval width ratio (between the equal- *α* split confidence interval and W-optimal interval) varies with the extent of the between-study heterogeneity. We see that the biggest gains from presenting the W-optimal interval as a confidence interval are when this heterogeneity is small, which is consistent with previous findings. Figure 5 also shows that the average optimal choice of $\alpha ^{*}_{2}$ is always between 4.5 % and 5 %, which again is consistent with our other results.

**Fig. 6:**
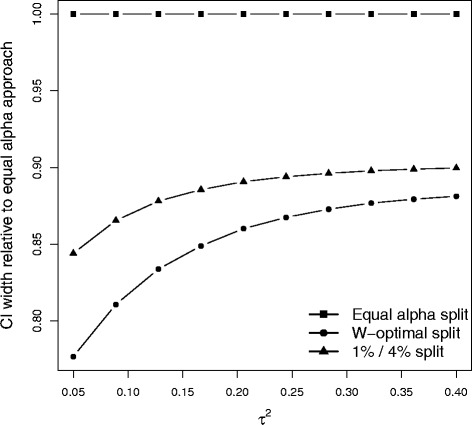
Mean width ratio (compared to the equal *α* approach) of the W-optimal and 0.01:0.04 split confidence intervals, as a function of *τ* ^2^

Figure 6 shows the estimated reduction in mean confidence interval width of the W-optimal interval and the *α*_1_=0.01,*α*_2_=0.04 split confidence intervals, each compared to the conventional equal *α* split as a function of *τ*^2^. Clearly, a considerable average 95 % confidence interval width reduction can be obtained by choosing a fixed but unequal *α* split and the further improvement afforded by the W-optimal is quite modest. The *α*_1_=0.01,*α*_2_=0.04 split appears to be quite an attractive option given that it also achieves nominal coverage and is immune to the natural concerns that accompany presenting the W-optimal interval as a confidence interval.

#### Jackson’s method

Jackson [8] proposed the competing method to the Q profile method described above. Jackson [8] shows that, unless there is substantial heterogeneity present, some simple choices of *a*_*i*_ yield confidence intervals with a shorter width than the Q-profile approach.

Tables 3 and 4 show simulation study results (under the same data generating model for meta-analyses as in the previous section with *k*=15 studies but with different simulated datasets) the estimated average 95 % confidence interval for *τ*^2^ width and coverage of the Q-profile approach and Jackson’s generalised Q-statistic using Jackson’s proposal of *a*_*i*_=1/*σ*_*i*_. In general the performance of Jackson’s Generalised *Q* statistic is highly similar to that of the *Q*-profile approach. However, Jackson’s method tends to yield slightly narrower confidence intervals when the between-study heterogeneity is small. This is consistent with the findings reported in Jackson [8] who assumed a different distribution for the within-study variance. These simulation studies suggest that similar recommendations for values of *α*_1_ and *α*_2_ can be made for both the Q profile and Jackson’s method.

**Table 3:** Confidence interval width of the Q-profile and Jackson’s Generalised Q statistic approaches under equal, optimal and 0.01:0.04 split strategies; *k*=15

*τ* ^2^	*I* ^2^	CI width		CI width ratio (wrt equal *α*)			
		Equal *α*		*W*-opt		0.01:0.04 split	
		Q-profile	Gen-Q	Q-profile	Gen-Q	Q-profile	Gen-Q
0.05	28.49	0.305	0.296	0.777	0.778	0.844	0.846
0.09	39.93	0.393	0.385	0.811	0.811	0.865	0.867
0.13	48.82	0.478	0.470	0.834	0.834	0.878	0.879
0.17	55.47	0.558	0.551	0.849	0.849	0.886	0.887
0.21	60.85	0.638	0.633	0.860	0.861	0.891	0.892
0.24	65.21	0.718	0.714	0.868	0.868	0.894	0.895
0.28	68.60	0.796	0.793	0.873	0.874	0.896	0.897
0.32	71.40	0.873	0.871	0.877	0.878	0.898	0.899
0.36	73.77	0.949	0.949	0.879	0.880	0.899	0.900
0.40	75.74	1.030	1.030	0.881	0.882	0.900	0.900

**Table 4:** e and Jackson’s Generalised Q statistic approaches under equal, optimal and 0.01:0.04 split strategies; *k*=15

*τ* ^2^	*I* ^2^	95 CI coverage (%)					
		Equal *α*		*W*-opt		0.01:0.04 split	
		Q-profile	Gen-Q	Q-profile	Gen-Q	Q-profile	Gen-Q
0.05	28.49	94.90	94.90	94.60	94.58	94.95	94.98
0.09	39.93	95.04	95.03	94.67	94.68	95.07	95.11
0.13	48.82	94.85	94.88	94.57	94.56	94.88	94.90
0.17	55.47	94.79	94.82	94.53	94.56	94.86	94.84
0.21	60.85	95.04	95.04	94.55	94.59	94.97	95.03
0.24	65.21	94.94	94.94	94.70	94.74	95.03	94.99
0.28	68.60	94.90	94.85	94.71	94.71	95.02	95.02
0.32	71.40	94.99	95.14	94.64	94.64	94.95	94.98
0.36	73.77	94.91	94.93	94.62	94.61	94.89	94.95
0.40	75.74	94.87	94.92	94.69	94.62	94.95	94.86

#### Conclusions from the simulation study

To summarise the findings from the simulation study, we find that considerably shorter 95 % confidence intervals for *τ*^2^ can be obtained by using *α*_2_>>*α*_1_. Jackson’s method appears to respond to the use of unconventional choices of *α*_1_ and *α*_2_ in a similar way to the Q profile method. Hence we suggest that the same conventions be used for all methods based on generalised heterogeneity statistics. We conclude that the W-optimal interval appears to have satisfactory coverage probabilities, despite theoretical objections and the natural concerns that accompany it, and its use as a confidence interval deserves further investigation and consideration.

### Sensitivity analyses for the average effect

**Fig. 7:**
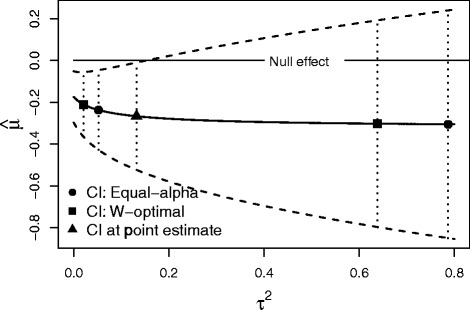
Sensitivity analysis for the average effect using the NSCLC4 data. The W-optimal interval provides a shorter confidence interval than the conventional approach

Although we regard shorter confidence intervals for the between-study variance as an important outcome in its own right, this can also be beneficial when making inferences about the average effect *μ*, which is usually the parameter of primary interest. For example, in small samples Jackson and Bowden [32] suggest using a sensitivity analysis, where we compute a confidence interval for *τ*^2^ and then apply the random-effects model to make inferences about the average effect using a range of values of *τ*^2^ that lie in this interval. The results using this procedure for the NSCLC4 data are shown in Fig. 7, where we show the range of results that are possible using the conventional ‘equal *α*’ (*α*_1_=*α*_2_=0.025) *Q* profile confidence interval and also the corresponding W-optimal interval. Graphical displays that are similar to this have previously been suggested [11, 33]. Using the DerSimonian and Laird point estimate of *τ*^2^ (see the triangular plotting points in Fig. 7) we infer a borderline statistically significant effect (indicating that the addition of chemotherapy is benefical) when using the conventional method, but this significance is lost when using the sensitivity analysis and either confidence interval for *τ*^2^. This is appropriate because the conventional method does not take into account the uncertainty in *τ*^2^, which is quite considerable. However the W-optimal interval, as a direct consequence of it being shorter and having a smaller upper bound, provides a reduced range of possible inferences for the average effect compared to the standard *Q* profile confidence interval. Since the estimated variance of the pooled effect is increasing in $\hat {\tau }^{2}$ under the random-effects model, we can anticipate that this will usually be the case. This illustrative analysis shows that making better inferences for one component of the random-effects model can have beneficial consequences when making inferences from all aspects of the the fitted model.

## Conclusions

Generalised heterogeneity statistics offer straightforward and direct ways of obtaining confidence intervals for the between-study variance parameter in a random-effects meta-analysis that have the correct coverage probability under the random-effects model even when the number of studies is small. However the resulting confidence intervals are usually very wide. We have found that assigning unequal proportions of the allowable type I error rate *α* to the lower and upper quantiles can dramatically reduce the width of resulting confidence intervals, enabling more precise inference. Given the potential gains in taking larger values of *α*_2_ to provide shorter confidence intervals, we present our results to the meta-analysis community and ask if larger values of *α*_2_ than the conventional 2.5 % should be used in application. Our motivation for investigating this, and our reasons for our recommendations below, are based upon our desire to reduce the width of confidence intervals without sacrificing their coverage probability. We have retained the conventional 95 % coverage probability because this is so enshrined in statistical practice but another way to justify using larger *α*_2_ is to present confidence intervals with a lower than conventional coverage probability; perhaps we should also defy this convention when presenting confidence intervals for *τ*^2^. Since *τ*^2^ is not usually of primary inferential interest this may also be acceptable to meta-analysts.

Meta-analysts should be aware that taking *α*_2_>0.025 and *α*_1_<0.025 results in smaller upper and lower confidence bounds than in the conventional *α*_1_=*α*_2_=0.025 interval. Hence our suggestion results in smaller *τ*^2^ being inferred. Given the extremely large values of *τ*^2^ that are often contained in conventional 95 % confidence intervals, which are usually extremely asymmetric around the point estimate, we feel that a modification that reduces this asymmetry and infers smaller *τ*^2^ is justifiable; see also our previous discussion.

Our results for confidence intervals for *τ*^2^ are not invariant to non-linear transformations. Hence we also produced some results for *τ*. *I*^2^ and *H*^2^ statistics can be conceptualised as functions of $\hat {\tau }^{2}$ and the within-study variances. Since the within-study variances are taken as fixed and known in the random effects model, a ‘true’ *I*^2^ and *H*^2^ can be taken to be the corresponding function evaluated at the true *τ*^2^ for which confidence intervals can be obtained. We have not however investigated the use of unequal *α* splits when calculating confidence intervals for *I*^2^ or *H*^2^ because in general they are used as descriptive rather than inferential statistics by the meta-analysis community.

The methods that we have presented are exact under the random-effects model but are only approximate when applied to real data, such as the four examples that we use here. This is because the random-effects model, as with any other statistical model, in general only provides an approximation when applied to real data. In particular the random-effects model takes the within-study variances as fixed and known and these can be quite imprecisely estimated in practice. This means that the random-effects model, and so the methods used here, can be quite a crude approximation when applied to real data. Kulinskaya and colleagues [34, 35] show that the distribution of quadratic forms in meta-analysis, when applied to real data, differ from their theoretical distributions under the random-effects model. We regard this as a serious problem only when the studies are small, although this can quite often be the case in application. Hence it is important to recognise that the methods presented here will rarely, if ever, be exact in application. Our motivating examples involve estimated log hazard ratios, for which it is hard to motivate the use of alternative distributional assumptions, but these examples are subject to these same concerns nonetheless. We investigated the use of methods based on generalised heterogeneity statistics for the reason described in the introduction, but see the recent and very thorough review by Veroniki et al. [36] for a description of both these and alternative methods for making inferences about the magnitude of *τ*^2^.

The confidence intervals are justified by the inversion of hypothesis tests and a further issue is that the use of *α*_1_≠*α*_2_ means that we are inverting an unusual and unconventional type of hypothesis test. Some type of special consideration would be needed to justify hypothesis tests of this type and our use of *α*_1_≠*α*_2_ is likely to appear curious to those who interpret confidence intervals in terms of their tautology with hypothesis testing, where confidence intervals’ primary purpose is to describe the parameter values that the hypothesis test does not reject. The use of equal tails when computing confidence intervals means that the confidence interval is based upon inverting a conventional two tailed hypothesis test, which eases interpretation because the tautology between hypothesis testing and confidence intervals is then especially strong and clear; we suspect that this is a main reason why equal tails are conventional when computing confidence intervals. We however are content to present confidence intervals with *α*_1_≠*α*_2_ that provide the nominal coverage probability and take confidence intervals’ primary purpose to cover the unknown true parameter with this probability. Applied analysts who conceptualise confidence intervals in terms of their coverage probability in repeated sampling, rather than primarily in terms of their tautology with hypothesis testing, should have little conceptual difficulty in using confidence intervals that use unequal tails.

Despite this, there is a further subtle point that should not be neglected. The usual hypothesis test for the presence of heterogeneity is a one-tailed test, where we reject the null hypothesis that the study effects are homogeneous if *Q*(0) is greater than $\chi ^{2}_{1-\alpha, k-1}$; *Q*(0) is equivalent to adopting the weights $w_{i}({\sigma _{i}^{2}}, \tau ^{2}) =1/{\sigma _{i}^{2}} $. In principle one could also test for extreme homogeneity [37] by instead concluding the data are highly homogenous if *Q*(0) is less than $\chi ^{2}_{\alpha, k-1}$. The conclusions from this pair of hypothesis tests will be ensured to be consistent with the conclusions from the Q profile confidence interval (for example, the null hypothesis that *τ*^2^=0 is rejected by the hypothesis test and *τ*^2^=0 does not lie in the confidence interval) if we take *α*_1_=*α*_2_=*α*. We could therefore perform the two hypothesis tests at the conventional 5 % significance level, and also calculate an equal tailed 90 % Q profile confidence interval, to ensure consistent conclusions. However alternative Q profile confidence intervals, such as a 95 % confidence interval, or a 90 % confidence interval with unequal tails, may or may not produce consistent conclusions with the two hypothesis tests. More generally, in order to ensure consistent conclusions for Q profile confidence intervals with unequal tails and this pair of hypothesis tests, we must use *α*_1_ as the significance level of the conventional hypothesis test for the presence of heterogeneity and *α*_2_ as the significance level for the hypothesis test for extreme homogeneity. Similar comments also apply when Jackson’s method is applied with the weights $w_{i}({\sigma _{i}^{2}}, \tau ^{2}) =1/{\sigma _{i}^{2}} $. However no such consistency with the conventional hypothesis tests described above is ensured when using Jackson’s method with alternative weights, including the proposed $w_{i}({\sigma _{i}^{2}}, \tau ^{2}) =1/\sigma _{i} $. To ensure consistency for the two types of hypothesis test and the confidence interval using Jackson’s method, the same set of weights would need to be used in all *Q* statistics *and* equivalent significance levels and tail probabilities would have to be adopted.

Our simulation study suggests that expressly presenting the W-optimal interval as a confidence interval only results in coverage probabilities that are very slightly less than the nominal level. Hence the use of the W-optimal interval as a confidence interval warrants further investigation, especially in situations where the number of studies is small. However it would be important to make it clear that the use of the W-optimal interval as a confidence interval had been chosen prior to analysis. However, a pre-specified unequal *α*-split, such as *α*_1_=0.01 and *α*_2_=0.04, can realise considerable average 95 % confidence interval width reductions whilst retaining the nominal coverage probability under the random-effects model. As a concrete recommendation we suggest that, if the reader is persuaded by the case for using unequal tail probabilities when using the methods we investigate here, then they should use the *α*_1_=0.01 and *α*_2_=0.04 split to obtain 95 % confidence intervals. If the repeated sampling properties of the *W*-optimal interval are confirmed to be satisfactory in future simulation studies and analytical work then this would become our recommended approach, but at this stage we wish to remain cautious in this regard.

To summarise, we suggest that the meta-analysis community should consider the case for presenting confidence intervals for *τ*^2^ with *α*_2_>*α*_1_ in the light of the results that we present. In the web Additional file 2 we present R code (the “AlphaPlot” function) that produces a figure like that of Fig. 2 for an arbitrary dataset, so that analysts can visualise the asymmetry of the *Q* profile statistic, the implications of using unequal tail probabilities and also the *W* optimal interval.

## Additional files

Additional file 1 Analytical investigation. (PDF 177 kb)

Additional file 2 R code for the function “AlphaPlot”. R code and data. (DOCX 19 kb)
