# Supplementary material for: Confidence intervals for the between-study variance in random-effects meta-analysis using generalised heterogeneity statistics: should we use unequal tails?
Source: BMC Med Res Methodol. 2016 Sep 7;16(1):118. doi: 10.1186/s12874-016-0219-y (PMC5015418; doi:10.1186/s12874-016-0219-y)
Supplement: Additional file 1 — Analytical investigation. (PDF 177 kb) [file 12874_2016_219_MOESM1_ESM.pdf]

Supplementary Materials

Dan Jackson and Jack Bowden

July 20, 2016

1 The analysis of the special case where all studies are the same 'size'

When $\sigma_i^2 = \sigma^2$ for all i, all the methods based on generalised heterogeneity statistics, and described in the main paper, become the same. This is because when all variances are the same, and so the y_i are independently and identically distributed, the only natural weights to use in DerSimonian and Kacker's generalised heterogeneity statistics are $a_i = 1$ for all i (i.e. use an unweighted heterogeneity statistic). These weights are proportional to those used in the conventional heterogeneity statistic $w_i = 1/\sigma_i^2 = 1/\sigma^2$ which therefore also becomes an unweighted heterogeneity statistic. Furthermore the Q profile method is also based on the unweighted heterogeneity statistic in this special case. Hence all the methods, unless unnatural weights were used in conjunction with DerSimonian and Kacker's heterogeneity statistic, use an unweighted sum of squares in the same way, and so give the same confidence interval for given values of α_1 and α_2. We will however present our analysis in terms of the Q profile method, because it is conceptually the most simple.

We will write $Q(\tau^2)$ as the Q profile pivot, ie we take $w_i(\sigma_i^2, \tau^2) = 1/(\sigma_i^2 + \tau^2)$, where we emphasise the dependence of this pivot on τ^2. It is convenient to write $X = Q(\tau^2) = \sigma^2 Q(0)/(\sigma^2 + \tau^2) = \sum_{i=1}^{k}(y_i - \bar{y})^2/(\sigma^2 + \tau^2) \sim \chi^2_{k-1}$.

1.1 Confidence intervals for the between-study variance, τ^2

There are three distinct types of confidence intervals for τ^2 when using the Q profile method: an empty set, a non-empty set where the lower confidence bound is truncated to zero and a non-empty confidence set where both the lower and upper bounds are positive. Firstly if $Q(0) < \chi^2_{\alpha_2,k-1}$, which is equivalent to $X < b_2$, where $b_2 = (1 - I^2)\chi^2_{\alpha_2,k-1}$, then an empty or [0,0]

confidence set is obtained; the width of the confidence set is zero under under either convention. Secondly if $\chi^2_{\alpha_2,k-1} < Q(0) < \chi^2_{1-\alpha_1,k-1}$, which is equivalent to $b_2 < X < b_1$, where $b_1 = (1-I^2)\chi^2_{1-\alpha_1,k-1}$, then the lower bound is truncated to zero and the upper bound, τ_u^2, is the solution of $Q(\tau_u^2) = \chi^2_{\alpha_2,k-1}$. This means that that the *ratio of the width of the confidence interval (τ_u^2) and the within-study variance* σ^2 is $b_2^{-1}X - 1$. And finally if $Q(0) > \chi^2_{1-\alpha_1,k-1}$, which is equivalent to $X > b_1$, then the lower and upper bounds are the solutions of $Q(\tau_l^2) = \chi^2_{1-\alpha_1,k-1}$ and $Q(\tau_u^2) = \chi^2_{\alpha_2,k-1}$ respectively, so that the ratio of the width of the confidence interval $(\tau_u^2 - \tau_l^2)$ and the within-study variance σ^2 is $(b_2^{-1} - b_1^{-1})X$.

Hence ratio of the expected width of the confidence interval and the within-study variance σ^2, w, is a function X given by

$$w(X) = \begin{cases} 0 & \text{if } X < b_2 \\ b_2^{-1}X - 1 & \text{if } b_2 < X < b_1 \\ (b_2^{-1} - b_1^{-1})X & \text{if } X > b_1 \end{cases} \tag{1}$$

The expected ratio of the width of the confidence interval and σ^2 is therefore

$$\mathrm{E}[w(X)] = \int_0^\infty w(x) f_{k-1}(x)\mathrm{d}x. \tag{2}$$

where $f_v(\cdot)$ is the probability density function of the χ^2 distribution with v degrees of freedom. By substituting (1) in into (2) we can evaluate the integral using the result that

$$\int_a^\infty x f_v(x) = v S_{v/2+1}(a/2) \tag{3}$$

where $S_a(\cdot)$ is the Survival function of the standard (scale parameter of one) Gamma distribution with shape parameter equal to a; this result can be obtained by making the substitution $u = x/2$ and a little manipulation. The use of this result quickly results in

$$\mathrm{E}[w(X)] = b_2^{-1}(k-1)S_{(k+1)/2}(b_2/2) + F_{k-1}(b_2) - b_1^{-1}(k-1)S_{(k+1)/2}(b_1/2) - F_{k-1}(b_1) \tag{4}$$

where $F_v(\cdot)$ is the cumulative distribution function for the χ^2 distribution with v degrees of freedom, $S_a(\cdot)$ is the Survival function of the standard (scale parameter of one) Gamma distribution with shape parameter equal to a, $b_1 = (1-I^2)\chi^2_{1-\alpha_1,k-1}$ and $b_2 = (1-I^2)\chi^2_{\alpha_2,k-1}$. Since values of both

$S_a(\cdot)$ and $F_v(\cdot)$ are produced routinely by standard statistical statistical software, (4) is very easily evaluated in order to give an indication of the width of confidence intervals from all methods that use generalised heterogeneity statistics.

Since $\mathrm{E}[w(X)]$ is the ratio of the expected width of the confidence interval and the within-study variance, multiplying this expression by σ^2 gives the expected length of the confidence interval. However the quantity in (4) is a convenient quantity to consider because it removes the need to specify the study size when assessing the implications of adopting different values of α_1 and α_2.

1.2 Confidence intervals for for the between-study standard error, τ

The confidence interval bounds of the confidence interval for τ are the square root of those of the confidence interval for τ^2. Hence, now using $w(X)$ to denote the expected width of the confidence interval for τ and the within-study standard deviation σ, equation (1) becomes

$$w(X) = \begin{cases} 0 & \text{if } X < b_2 \\ \sqrt{b_2^{-1}X - 1} & \text{if } b_2 < X < b_1 \\ \sqrt{b_2^{-1}X - 1} - \sqrt{b_1^{-1}X - 1} & \text{if } X > b_1 \end{cases} \quad (5)$$

The expectation of $w(X)$ is as given in (2) with $w(X)$ as given in (5). The necessary integrals can then be obtained numerically, where to improve the stability of the numerical methods we evaluated the integrals as

$$\int_a^b \sqrt{c^{-1}x - 1} f_{k-1}(x)\mathrm{d}x = c\int_{a/c-1}^{b/c-1} \sqrt{x} f_{k-1}(c(x+1))\mathrm{d}x$$

2 Results

We will use equation (4) to consider four cases: the conventional use of 95% confidence intervals with equal tails ($\alpha_2 = 0.025$), two uses of unequal tails ($\alpha_2 = 0.03, 0.04$) and a one-sided confidence interval ($\alpha_2 = 0.05$). The values shown in Table 1 are the expected width of these confidence intervals in multiples of σ^2. We show results for $I^2 = 0, 0.15, 0.3, 0.5, 0.75, 0.9$ in order to cover a very wide range of possibilities. Given the empirical results in the main paper, we anticipate that $\alpha_2 = 0.04, 0.05$ will result in shorter

confidence intervals than the more equal tails that use $\alpha_2 = 0.025, 0.03$. In Table 1 we show some results for inordinately large sample sizes to see what happens asymptotically. From Table 1 we can see that $\alpha_2 = 0.04$ consistently results in shorter confidence intervals unless k or I^2 are very large, in which case a more equal allocation of the α spending becomes preferable. For the type of fairly typical meta-analyses described in the main paper ($k < 20$, I^2 around 50%) we can see further evidence in Table 1 that one-sided confidence intervals ($\alpha_2 = 0.05$) are, on average, the shortest. However for larger k and I^2, the rather extreme case of $\alpha_2 = 0.05$ is not optimal. This makes sense because in large samples τ^2 is more precisely estimated and for large I^2 we need to spend some of α in ruling out small values of τ^2 in order to reduce the average confidence interval width. Thus, this analytical investigation provides further evidence that we should consider using $\alpha_2 >> \alpha_1$ but also that extreme cases like $\alpha_2 = 0.05$ may not be appropriate despite the empirical evidence from the CERVIX3 and NSCLC1 examples.

In Table 2 we show the results as in Table 1 but this time we report our findings on the τ scale. Note that in Table 2 the average length of 95% confidence intervals for τ is shown in multiples of σ whereas in Table 1 the average length of 95% confidence intervals for τ^2 is shown in multiples of σ^2. Table 2 confirms that taking $\alpha_2 > \alpha_1$ can result in shorter confidence intervals in similar situations as before. However the use of $\alpha_2 = 0.05$ looks less promising in Table 2 than in Table 1 and there appears to be less benefit in using $\alpha_2 > \alpha_1$ when working on the between-study standard error scale. Despite this, for $k = 10$, we find that notably shorter confidence intervals for τ can still be obtained by using unequal tails, which supports our empirical investigation. Since meta-analyses with few studies ($k < 10$, say) are commonplace, we conclude that notably shorter confidence intervals for both τ and τ^2 can be obtained in practice by taking $\alpha_2 > \alpha_1$. Taking $\alpha = 0.04$ in particular consistently produces produces shorter 95% confidence intervals than $\alpha = 0.025$ in small samples.

2.1 A comparison between the results in Tables 1 and 2

For $k = 10$, all the average confidence interval widths in Table 1 are greater than those in Table 2. This is because the upper confidence interval bounds for τ^2, and so the average confidence interval widths, are often much greater than σ^2 in small samples. Hence, because the the square root function more dramatically reduces large values, the upper confidence bounds (and so the average confidence interval widths) on the τ scale are generally reduced by

Table 1: The average width of 95% confidence intervals for τ^2 obtained using generalised heterogeneity statistics when $\sigma_i^2 = \sigma^2$ for all i. The results tabulated are these widths in multiples of σ^2, so that this table shows values of (4). For each combination of k and I^2, the ordered quadrouple shows the results using $\alpha_2 = 0.025, 0.03, 0.04, 0.05$, where $\alpha_1 + \alpha_2 = 0.05$.

	I^2					
k	0	0.15	0.3	0.5	0.75	0.9
10	(2.33, 2.16, 1.91, 1.72)	(2.91, 2.71, 2.41, 2.19)	(3.73, 3.49, 3.13, 2.87)	(5.51, 5.18, 4.71, 4.41)	(11.40, 10.77, 9.88, 9.83)	(28.60, 27.02, 24.83, 26.07)
20	(1.13, 1.07, 0.96, 0.89)	(1.50, 1.42, 1.30, 1.21)	(2.00, 1.91, 1.77, 1.68)	(3.03, 2.92, 2.76, 2.76)	(6.22, 6.00, 5.73, 6.51)	(15.55, 15.01, 14.34, 17.78)
30	(0.81, 0.76, 0.70, 0.64)	(1.11, 1.06, 0.99, 0.93)	(1.52, 1.47, 1.39, 1.34)	(2.31, 2.25, 2.16, 2.28)	(4.69, 4.57, 4.43, 5.55)	(11.73, 11.41, 11.07, 15.38)
50	(0.55, 0.53, 0.48, 0.45)	(0.81, 0.78, 0.73, 0.70)	(1.14, 1.11, 1.07, 1.06)	(1.70, 1.67, 1.64, 1.89)	(3.42, 3.35, 3.30, 4.78)	(8.55, 8.38, 8.25, 13.44)
100	(0.35, 0.33, 0.31, 0.29)	(0.57, 0.55, 0.53, 0.51)	(0.80, 0.79, 0.78, 0.84)	(1.16, 1.14, 1.14, 1.57)	(2.31, 2.29, 2.28, 4.14)	(5.79, 5.71, 5.71, 11.85)
500	(0.14, 0.13, 0.12, 0.11)	(0.28, 0.28, 0.28, 0.31)	(0.36, 0.36, 0.36, 0.59)	(0.50, 0.50, 0.51, 1.23)	(1.00, 1.00, 1.02, 3.45)	(2.50, 2.49, 2.54, 10.13)
1000	(0.09, 0.09, 0.08, 0.08)	(0.21, 0.21, 0.21, 0.27)	(0.25, 0.25, 0.26, 0.54)	(0.35, 0.35, 0.36, 1.16)	(0.70, 0.70, 0.72, 3.31)	(1.76, 1.76, 1.80, 9.78)

Table 2: The average width of 95% confidence intervals for τ obtained using generalised Cochran statistics when $\sigma_i^2 = \sigma^2$ for all i. The results tabulated are these widths in multiples of σ. For each combination of k and I^2, the ordered quadrouple shows the results using $\alpha_2 = 0.025, 0.03, 0.04, 0.05$, where $\alpha_1 + \alpha_2 = 0.05$.

	I^2					
k	0	0.15	0.3	0.5	0.75	0.9
10	(1.42, 1.36, 1.27, 1.20)	(1.59, 1.53, 1.44, 1.38)	(1.78, 1.72, 1.64, 1.60)	(2.06, 2.01, 1.95, 2.01)	(2.57, 2.51, 2.45, 3.03)	(3.68, 3.57, 3.45, 4.95)
20	(1.00, 0.96, 0.91, 0.87)	(1.15, 1.12, 1.07, 1.04)	(1.29, 1.27, 1.24, 1.25)	(1.41, 1.40, 1.40, 1.62)	(1.61, 1.58, 1.58, 2.51)	(2.30, 2.26, 2.23, 4.15)
30	(0.84, 0.82, 0.78, 0.74)	(0.99, 0.97, 0.94, 0.92)	(1.10, 1.09, 1.08, 1.12)	(1.14, 1.14, 1.16, 1.48)	(1.26, 1.24, 1.25, 2.33)	(1.81, 1.78, 1.78, 3.88)
50	(0.70, 0.68, 0.65, 0.62)	(0.84, 0.83, 0.81, 0.81)	(0.91, 0.91, 0.92, 1.01)	(0.85, 0.85, 0.88, 1.36)	(0.95, 0.94, 0.95, 2.17)	(1.36, 1.35, 1.36, 3.64)
100	(0.56, 0.54, 0.52, 0.50)	(0.69, 0.69, 0.68, 0.70)	(0.67, 0.68, 0.71, 0.90)	(0.58, 0.58, 0.60, 1.24)	(0.65, 0.65, 0.66, 2.03)	(0.94, 0.94, 0.95, 3.43)
500	(0.35, 0.34, 0.33, 0.32)	(0.39, 0.40, 0.42, 0.55)	(0.28, 0.28, 0.29, 0.77)	(0.25, 0.25, 0.26, 1.11)	(0.29, 0.29, 0.30, 1.86)	(0.41, 0.41, 0.43, 3.18)
1000	(0.29, 0.28, 0.27, 0.26)	(0.27, 0.27, 0.29, 0.52)	(0.19, 0.19, 0.20, 0.73)	(0.18, 0.18, 0.18, 1.07)	(0.20, 0.20, 0.21, 1.82)	(0.29, 0.29, 0.30, 3.13)

this transformation when k is small.

However for very large k we can see that larger values are obtained in Table 2 than in Table 1 when $I^2 < 0.5$ but also that this is reversed when $I^2 > 0.5$. To understand why this is so, since Tables 1 and 2 show average widths in multiples of σ^2 and σ respectively, to simplify the argument take $\sigma = \sigma^2 = 1$. Then $I^2 < 0.5$ is equivalent to $\tau^2 < 1$, which means that in large samples the upper confidence bounds for τ^2 are also likely to be less than one; when we square root these upper confidence interval bounds to obtain upper confidence interval bounds on the τ scale these bounds become bigger. Hence larger average confidence interval widths on the τ scale can be obtained. However $I^2 > 0.5$ is equivalent to $\tau^2 > 1$, so that the average upper confidence interval bounds for τ^2 are also greater than one; when we square root these confidence bounds to obtain confidence bounds on the τ scale these bounds become smaller, which results in smaller average confidence interval widths.

For those who might wish to further reconcile the results in Tables 1 and 2, if we denote a value in Table 2 as c_2, and the corresponding expected lower and upper confidence interval bounds as L and U, then we have $U - L = c_2$. Then the large sample (here we need large k) approximation $E[X^2] \approx E[X]^2$ gives $U^2 - L^2 \approx c_1$, where c_1 is the corresponding value in Table 1. Upon solving these two simultaneous equations for L and U, we obtain $L \approx (c_1/c_2 - c_2)/2$ and $U \approx (c_1/c_2 + c_2)/2$. For example, for $k = 1000$, $I^2 = 0$ and $\alpha_2 = 0.025$, from Tables 1 and 2 we have $c_1 = 0.09$ and $c_2 = 0.29$ which gives $L = 0.01$ and $U = 0.30$. This indicates that we are able to quite accurately estimate the true value of $\tau^2 = 0$. However for $k = 1000$, $I^2 = 0.9$ and $\alpha_2 = 0.025$, from Tables 1 and 2 we have $c_1 = 1.76$ and $c_2 = 0.29$ which gives $L = 2.89$ and $U = 3.18$. This means that we are quite accurately able to estimate the true value of $\tau = 3$ (when $\sigma^2 = 1$, $I^2 = 0.9$ is equivalent to $\tau^2 = 9$). The use of further c_1 and c_2 from Tables 1 and 2, whilst assuming that $k \geq 100$ so that the large sample approximation above is accurate enough for use in calculation, reconciles the large sample results in Tables 1 and 2.
