# Supplementary material for: Confidence intervals for the between-study variance in random-effects meta-analysis using generalised heterogeneity statistics: should we use unequal tails?
Source: BMC Med Res Methodol. 2016 Sep 7;16(1):118. doi: 10.1186/s12874-016-0219-y (PMC5015418; doi:10.1186/s12874-016-0219-y)
Supplement: Additional file 2 — R code for the function “AlphaPlot”. R code and data. (DOCX 19 kb) [file 12874_2016_219_MOESM2_ESM.docx]

**R code for the function “AlphaPlot”: This is a function of the study outcomes (y) and the within-study standard errors (s). If you have within-study variances don’t forget to square root them to get s! This function produces a plot like that in Figure 2 of the paper for an arbitrary dataset. This function is intended to help meta-analysts visualise the implications of using unequal tails when using the Q profile method to calculate 95% confidence intervals. Please try it on your examples!**

AlphaPlot = function(y,s){

# Requires functions PM(), Alphafind() and PM_alpha() #

# PM()

k = length(y)

PM = function(y = y, s = s, Alpha = 0.1){

K = length(y) ; sig = qnorm(1-Alpha/2)

df = K-1

low = qchisq((Alpha/2), df) ; up = qchisq(1-(Alpha/2), df)

Quant = c(low,df,up) ; L = length(Quant)

Tausq = NULL ; Isq = NULL

CI = matrix(nrow = L, ncol = 2) ;MU = NULL

v = 1/s^2 ; sum.v = sum(v) ; typS = sum(v*

(K-1))/(sum.v^2 - sum(v^2))

for(j in 1:L){

tausq = 0 ; F = 1 ;TAUsq = NULL

while(F>0){

TAUsq = c(TAUsq, tausq)

w = 1/(s^2+tausq) ; sum.w = sum(w) ; w2 = w^2

yW = sum(y*w)/sum.w ; Q1 = sum(w*(y-yW)

^2)

Q2 = sum(w2*(y-yW)^2) ; F = Q1-Quant[j]

Ftau = max(F,0) ; delta = F/Q2

tausq = tausq + delta

}

MU[j] = yW ; V = 1/sum(w)

Tausq[j] = max(tausq,0) ; Isq[j] = Tausq[j]/(Tausq[j]+typS)

CI[j,] = yW + sig*c(-1,1) *sqrt(V)

}

return(list(tausq = Tausq, muhat = MU,

Isq = Isq, CI = CI, quant = Quant))

}

Standard = PM(y,s,Alpha = 0.05)

# Alphafind()

Alphafind = function(a){

# choose initial value of

# alpha1

alpha1 = a[1]

low = qchisq(alpha1, df)

alpha2 = Alpha - alpha1

high = qchisq(1-alpha2, df)

Quant = c(low,high) ; L = length(Quant)

Tausq = NULL

for(j in 1:L){

tausq = 0 ; F = 1 ;TAUsq = NULL

while(F>0){

TAUsq = c(TAUsq, tausq)

w = 1/(s^2+tausq) ; sum.w = sum(w) ; w2 = w^2

yW = sum(y*w)/sum.w ; Q1 = sum(w*(y-yW)

^2)

Q2 = sum(w2*(y-yW)^2) ; F = Q1-Quant[j]

Ftau = max(F,0) ; delta = F/Q2

tausq = tausq + delta

}

Tausq[j] = max(tausq,0)

}

Diff = abs(Tausq[1]-Tausq[2])

}

# PM_alpha()

PM_alpha = function(y = y, s = s, alpha1=0.025,alpha2=0.025){

K = length(y) ;

df = K-1

low = qchisq(alpha1,df) ; up = qchisq(1-alpha2, df)

Quant = c(low,df,up) ; L = length(Quant)

Tausq = NULL

for(j in 1:L){

tausq = 0 ; F = 1 ;TAUsq = NULL

while(F>0){

TAUsq = c(TAUsq, tausq)

w = 1/(s^2+tausq) ; sum.w = sum(w) ; w2 = w^2

yW = sum(y*w)/sum.w ; Q1 = sum(w*(y-yW)^2)

Q2 = sum(w2*(y-yW)^2) ; F = Q1-Quant[j]

Ftau = max(F,0) ; delta = F/Q2

tausq = tausq + delta

}

Tausq[j] = max(tausq,0) }

return(list(tausq = Tausq, quant = Quant))

}

# Find optimal alpha split

Alpha = 0.05

K = length(y)

df = K-1

results = optimise(Alphafind,c(0,Alpha))

alpha1 = results$min

alpha2 = Alpha - results$min

results2 = PM_alpha(y = y, s = s,

alpha1=alpha1,alpha2=alpha2)

Ub = ceiling(qchisq(1-alpha2,k-1))

Tau2 = NULL

VV = seq(1,Ub)

for(i in 1:length(VV)){

# Find tausqU

Quant = VV[i]

tausq = 0 ; F = 1 ;TAUsq = NULL

while(F>0){

TAUsq = c(TAUsq, tausq)

w = 1/(s^2+tausq) ; sum.w = sum(w) ; w2 = w^2

yW = sum(y*w)/sum.w ; Q1 = sum(w*(y-yW)

^2)

Q2 = sum(w2*(y-yW)^2) ; F = Q1-Quant

Ftau = max(F,0) ; delta = F/Q2

tausq = tausq + delta

}

Tau2 = c(Tau2,max(tausq,0))

}

##############

# start plot #

##############

k = length(y)

xx = seq(0,Ub,length=1000) # approx 3 times as large as k

yy = dchisq(xx,k-1)

plot(xx,yy,type="l",lwd=3,ylab="Density f(x)",xlab="",main="",cex.lab=1.2,axes=FALSE)

value = seq(0,Ub) # also in relation to k

Value = seq(0,Ub) # also in relation to k

axis(1,at=value,line=2)

axis(1,at=Value,100*round(pchisq(Value,k-1),2),line=0)

axis(2,at=c(0,0.02,0.04,0.06,0.08,0.1),line=0)

# Find optimal alpha split

axis(3,at=seq(1,Ub),round(Tau2,2),line=0)

points(qchisq(alpha1,k-1),dchisq(qchisq(alpha1,k-1),k-1),pch=19,cex=2,col="red")

points(qchisq(1-alpha2,k-1),dchisq(qchisq(1-alpha2,k-1),k-1),pch=19,cex=2,col="red")

points(qchisq(0.025,k-1),dchisq(qchisq(0.025,k-1),k-1),pch=19,cex=2)

points(qchisq(0.975,k-1),dchisq(qchisq(0.975,k-1),k-1),pch=19,cex=2)

lines(rep(qchisq(alpha1,k-1),2),c(-1,1),lty=2,col="red")

lines(rep(qchisq(1-alpha2,k-1),2),c(-1,1),lty=2,col="red")

lines(rep(qchisq(0.025,k-1),2),c(-1,1),lty=2,col="black")

lines(rep(qchisq(0.975,k-1),2),c(-1,1),lty=2,col="black")

tausq.CI = c(results2$tausq[3],results2$tausq[1])

tausq.CI_original = c(Standard$tausq[3],Standard$tausq[1])

print("Standard Q-profile 95% CI for tausq (black dots)")

print(tausq.CI_original)

print("Minimum width Q-profile 95% CI for tausq (red dots)")

print(tausq.CI)

}

**R code to produce figure 2: This code does not all generalise to other datasets.**

rm(list=ls(all=TRUE))

#################################

# R code to re-produce Figure 2 #

# for the NSCLC4 data set #

##################################

# Requires modification to apply #

# to new data #

##################################

# Requires functions PM(), Alphafind() and PM_alpha() #

# PM()

PM = function(y = y, s = s, Alpha = 0.1){

K = length(y) ; sig = qnorm(1-Alpha/2)

df = K-1

low = qchisq((Alpha/2), df) ; up = qchisq(1-(Alpha/2), df)

Quant = c(low,df,up) ; L = length(Quant)

Tausq = NULL ; Isq = NULL

CI = matrix(nrow = L, ncol = 2) ;MU = NULL

v = 1/s^2 ; sum.v = sum(v) ; typS = sum(v*

(K-1))/(sum.v^2 - sum(v^2))

for(j in 1:L){

tausq = 0 ; F = 1 ;TAUsq = NULL

while(F>0){

TAUsq = c(TAUsq, tausq)

w = 1/(s^2+tausq) ; sum.w = sum(w) ; w2 = w^2

yW = sum(y*w)/sum.w ; Q1 = sum(w*(y-yW)

^2)

Q2 = sum(w2*(y-yW)^2) ; F = Q1-Quant[j]

Ftau = max(F,0) ; delta = F/Q2

tausq = tausq + delta

}

MU[j] = yW ; V = 1/sum(w)

Tausq[j] = max(tausq,0) ; Isq[j] = Tausq[j]/(Tausq[j]+typS)

CI[j,] = yW + sig*c(-1,1) *sqrt(V)

}

return(list(tausq = Tausq, muhat = MU,

Isq = Isq, CI = CI, quant = Quant))

}

# Alphafind()

Alphafind = function(a){

# choose initial value of

# alpha1

alpha1 = a[1]

low = qchisq(alpha1, df)

alpha2 = Alpha - alpha1

high = qchisq(1-alpha2, df)

Quant = c(low,high) ; L = length(Quant)

Tausq = NULL

for(j in 1:L){

tausq = 0 ; F = 1 ;TAUsq = NULL

while(F>0){

TAUsq = c(TAUsq, tausq)

w = 1/(s^2+tausq) ; sum.w = sum(w) ; w2 = w^2

yW = sum(y*w)/sum.w ; Q1 = sum(w*(y-yW)

^2)

Q2 = sum(w2*(y-yW)^2) ; F = Q1-Quant[j]

Ftau = max(F,0) ; delta = F/Q2

tausq = tausq + delta

}

Tausq[j] = max(tausq,0)

}

Diff = abs(Tausq[1]-Tausq[2])

}

# PM_alpha()

PM_alpha = function(y = y, s = s, alpha1=0.025,alpha2=0.025){

K = length(y) ;

df = K-1

low = qchisq(alpha1,df) ; up = qchisq(1-alpha2, df)

Quant = c(low,df,up) ; L = length(Quant)

Tausq = NULL

for(j in 1:L){

tausq = 0 ; F = 1 ;TAUsq = NULL

while(F>0){

TAUsq = c(TAUsq, tausq)

w = 1/(s^2+tausq) ; sum.w = sum(w) ; w2 = w^2

yW = sum(y*w)/sum.w ; Q1 = sum(w*(y-yW)^2)

Q2 = sum(w2*(y-yW)^2) ; F = Q1-Quant[j]

Ftau = max(F,0) ; delta = F/Q2

tausq = tausq + delta

}

Tausq[j] = max(tausq,0) }

return(list(tausq = Tausq, quant = Quant))

}

#####################

# supply data #

#############################

# y = effect size vector #

# s = standard error vector #

#############################

# e.g NSCLC4 data

y = c(0.3744292,-0.5481852,-0.1355263,-0.2018027,-0.3994334,0.1536424,

0.1565171,-0.3324157,-0.1848142,-0.7980820,-1.5915280)

s = c(0.1510995,0.3537746,0.1622214,0.1582326,0.1881775,0.3639373,0.2311251,

0.2623416,0.1797503,0.2308170,0.3889549)

# start plot

k = length(y)

xx = seq(1,30,length=1000) # approx 3 times as large as k

yy = dchisq(xx,k-1)

plot(xx,yy,type="l",lwd=3,ylab="Density f(x)",xlab="",main="",cex.lab=1.2,axes=F,

xlim=c(-2,30),ylim=c(-0.06,0.13))

value = c(1,5,10,15,20,25,30) # also in relation to k

Value = c(1,3.94,5,10,15,20,25,30) # also in relation to k

axis(1,at=value,line=-4)

axis(1,at=Value,100*round(pchisq(Value,k-1),2),line=-2)

axis(2,at=c(0,0.02,0.04,0.06,0.08,0.1),line=-2)

# Find optimal alpha split

Alpha = 0.05

df = k-1

results = optimise(Alphafind,c(0,Alpha))

alpha1 = results$min

alpha2 = Alpha - results$min

results2 = PM_alpha(y = y, s = s,

alpha1=alpha1,alpha2=alpha2)

# plot the CI width as a function of alpha_1

Tau2 = NULL

VV = seq(2,30,by=2)

for(i in 1:length(VV)){

# Find tausqU

Quant = VV[i]

tausq = 0 ; F = 1 ;TAUsq = NULL

while(F>0){

TAUsq = c(TAUsq, tausq)

w = 1/(s^2+tausq) ; sum.w = sum(w) ; w2 = w^2

yW = sum(y*w)/sum.w ; Q1 = sum(w*(y-yW)

^2)

Q2 = sum(w2*(y-yW)^2) ; F = Q1-Quant

Ftau = max(F,0) ; delta = F/Q2

tausq = tausq + delta

}

Tau2 = c(Tau2,max(tausq,0))

}

axis(3,at=seq(2,30,by=2),round(Tau2,2),line=-1)

# Find optimal alpha split

Alpha = 0.05

K = length(y)

df = K-1

results = optimise(Alphafind,c(0,Alpha))

alpha1 = results$min

alpha2 = Alpha - results$min

results2 = PM_alpha(y = y, s = s,

alpha1=alpha1,alpha2=alpha2)

points(qchisq(alpha1,k-1),dchisq(qchisq(alpha1,k-1),k-1),pch=19,cex=2,col="black")

points(qchisq(1-alpha2,k-1),dchisq(qchisq(1-alpha2,k-1),k-1),pch=19,cex=2,col="black")

points(qchisq(0.025,k-1),dchisq(qchisq(0.025,k-1),k-1),pch=19,cex=2)

points(qchisq(0.975,k-1),dchisq(qchisq(0.975,k-1),k-1),pch=19,cex=2)

lines(rep(qchisq(alpha1,k-1),2),c(-0.055,0.133),lty=2,col="black")

lines(rep(qchisq(1-alpha2,k-1),2),c(-0.055,0.133),lty=2,col="black")

lines(rep(qchisq(0.025,k-1),2),c(-0.055,0.133),lty=2,col="black")

lines(rep(qchisq(0.975,k-1),2),c(-0.055,0.133),lty=2,col="black")

######################################################

# Now expand plot window to at or near maximum size #

# before putting in legends below #

######################################################

legend(-1.3,-0.035,"x",bty="n",cex=1.3)

legend(-3,-0.05,expression(Pr(X <= x)),bty="n",cex=1.3)

legend(-3,0.143,expression(paste(hat(tau)^"2" == Q^-1,"(x)")),bty="n",cex=1.3)

# adjust legend input accordingly given values of

# alpha1 and alpha2 as below

legend(6,0.03,c("2.5th percentile:3.24", # 0.025, qchisq(0.025,k-1)

"4.8th percentile: 3.90", # alpha1, qchisq(alpha1,k-1)

"97.5th percentile: 20.48", # 1-0.025, qchisq(1-0.025,k-1)

"99.8th percentile: 28.91"),bty="n", # 1-alpha2, qchisq(1-alpha2,k-1)

pch=19,cex=1.2,col="black")
